# Preparation of Porous Ellipsoidal Bismuth Oxyhalide Microspheres and Their Photocatalytic Performances

**DOI:** 10.3390/ma15176035

**Published:** 2022-09-01

**Authors:** Bing Luo, Canfeng Wu, Fuzeng Zhang, Tingting Wang, Yingbang Yao

**Affiliations:** 1 China Southern Power Grid, Guangzhou 510623, China; 2 School of Materials and Energy, Guangdong University of Technology, Guangzhou 510006, China

**Keywords:** bismuth oxyhalides, photocatalytic degradation, holes, microspheres

## Abstract

Well-dispersed and uniform porous ellipsoidal-shaped bismuth oxyhalides (nominal composition: 80%BiOCl/20%BiOI) microspheres were obtained by a facile solvothermal method, in which process the use of polyvinylpyrrolidone (PVP) as template agent was found to be crucial. At 150 °C, elliptical porous particles with a particle size of 0.79 μm were formed. Instead of forming solid solutions, the study of X-ray diffraction (XRD) and high-resolution transmission electron microscopy (HRTEM) shows that the prepared 80%BiOCl/20%BiOI microspheres are composite of BiOCl and BiOI in nature and the obtained crystallite size is about 5.6 nm. The optical bandgap of 80%BiOCl/20%BiOI was measured to be 2.93 eV, which is between the bandgap values of BiOCl and BiOI. The 80%BiOCl/20%BiOI microspheres were able to decompose various organic dyes (rhodamine B-RhB, methyl orange-MO, methylene blue-MB, methyl violet-MV) under an illuminated condition with the degradation rate in the order of RhB > MB > MV > MO, and 98% of RhB can be degraded in 90 min. Radical scavenger tests showed that photogenerated holes are the main active species for the photocatalytic decomposition of all of the tested organic dyes. Our results show that the obtained porous ellipsoidal-shaped 80%BiOCl/20%BiOI microspheres are promising for the degradation of various organic pollutants under the illumination of visible light.

## 1. Introduction

The photocatalytic effect has been the focus of catalytic research for several decades [1]. It finds applications in various fields, such as the decomposition of organic pollutants [2], hydrogen generation through water splitting [3], reduction of CO_2_ [4] or NO_x_ [5], etc. In terms of pollutant treatment, photocatalytic treatment is considered an effective and environmentally friendly treatment method. Under UV or visible light irradiation, the pollutants are decomposed into carbon dioxide and water without secondary pollutants. With the development of nanotechnology, people have continuously improved the photocatalytic efficiency of materials by changing their microstructure [6,7]. In addition, semiconductor photocatalyst has the characteristics of stable performance, low cost, and long service life, which has become an effective means of treating organic dyes [8,9]. In early studies, photocatalytic reactions were mainly activated by the catalyst absorbed in the ultraviolet wavelength, such as TiO_2_ [10]. The biggest advantage of TiO_2_ is that it has a suitable flat band potential and high chemical stability. However, due to its large band gap energy, it can only absorb ultraviolet light and cannot absorb visible light. Moreover, it has a lower electron transfer rate to dissolved oxygen and a higher recombination rate of electron-hole pairs, which will affect the overall quantum yield, making the photocatalytic efficiency limited [11]. Nowadays, heterogeneous structures and chemical modification have been proposed to improve the charge transfer ability, and different photocatalytic efficiencies can be obtained by compounding different materials [9,12], various potential photocatalysts which can work with visible light have been developed with improved photocatalytic efficiencies [13,14,15,16].

In recent years, bismuth oxyhalides (BiOX, X = F, Cl, Br, I) start to attract the interests of researchers owing to their unique layer structure and special photocatalytic properties [17,18]. The crystal structure of BiOX is composed of a layer of [Bi_2_O_2_] slab interleaved by two slabs of halogen atoms. The valence band of BiOX is mainly formed by the O 2p and X np (n = 2–5 for X = F, Cl, Br, I) orbitals, while the conduction band is mainly composed of the Bi 6p orbital. Such configuration of band structure provides a potential opportunity for tuning the position of the valence band by replacing the X elements with different electronegativities. The bandgap of BiOX was found to decrease with the atomic number of X element, i.e., from 3.64 eV for BiOF, 3.22 eV for BiOCl, 2.64 eV for BiOBr to 1.77 eV for BiOI [17]. Based on the bandgap values, one can easily find that BiOBr and BiOI photocatalysts can be activated by visible light indicating their potential application as efficient photocatalysts. As a result, most of the current studies are focused on these two kinds of BiOX and various strategies are developed to enhance their photocatalytic performances. Benefits from the tunable band structures of BiOX, many researchers tried to identify the optimum X composition by alloying different X elements to form solid solutions, such as BiBr_x_I_1−x_ [18,19], BiCl_x_I_1−x_ [20], or BiCl_x_Br_1−x_ [21,22], etc. However, more work needs to be done before reaching a conclusion regarding which X composition is best for the most enhanced photocatalytic performance.

On the other hand, the diffuse light absorption ability and surface area are much influenced by the microstructure of BiOX, and hence the photocatalytic performance. Therefore, various attempts have been made to prepare nano-flakes or 3D hierarchical structures of BiOX [18,19,20,21,22]. Wang et al. used the ultrasonication-assisted hydrothermal method to synthesize BiOBr_x_I_1−x_ nanoplatelets with tunable bandgap and excellent photocatalytic performance [19]. Qing et al. prepared hierarchical flower-like BiOBr_x_I_1−x_ particles using the solvothermal method and found the photocatalytic activity under visible light illumination were improved due to the adoption of a hierarchical structure [15]. Chen et al. produced flower-like BiOCl_x_Br_1−x_ compounds and studied their capability for photocatalytic oxidation of NO under visible light [21]. These 3D hierarchical structures have the virtues of: (1) large surface area, which provides plenty of active sites for photocatalytic reactions; (2) unique porous structure, which facilitates the diffusion and transportation of reactants and products; (3) anti-agglomeration effect, which prohibits the aggregation of nanoparticles under various conditions; (4) large light absorption efficiency, which is benefited from the multiple light reflections inside the complex structure [23,24,25].

In this report, we report a facile solvothermal method to prepare 3D hierarchical porous ellipsoidal-shaped 80%BiOCl/20%BiOI particles, during which ethylene glycol (EG) was used as a solvent and polyvinyl pyrrolidone (PVP) was used as template agent. The band gap is known to play a crucial role in photocatalytic applications. And 80%BiOCl/20%BiOI has a suitable band gap for visible light activation [17]. Therefore, we chose this chemical composition. The unique 3D hierarchical microstructure combined with the specific composition of X (i.e., Cl_0.8_I_0.2_) provides the material with a high photocatalytic degradation efficiency towards various organic dyes, such as RhB, MO, MB, and MV.

## 2. Experimental

### 2.1. Materials

Bismuth nitride pentahydrate (Bi(NO_3_)_3_.5H_2_O, ≥99%), potassium chloride (KCl, 99.5%), potassium iodide (KI, ≥99%), PVP (K30 M.W.58,000), EG (98%) were used as starting raw materials. All of these materials were bought from Aladdin Co. (Shanghai, China) and used as received. Rhodamine B (RhB, AR), Methyl orange (MO, AR), Methylene blue (MB, AR), and Methyl violet (MV, AR) were purchased from Damao Trading Co. (Tianjin, China) and used as organic pollutants for evaluation of photocatalytic performance.

### 2.2. Synthesis of Ellipsoidal-Shaped BiOX

**Scheme 1:**
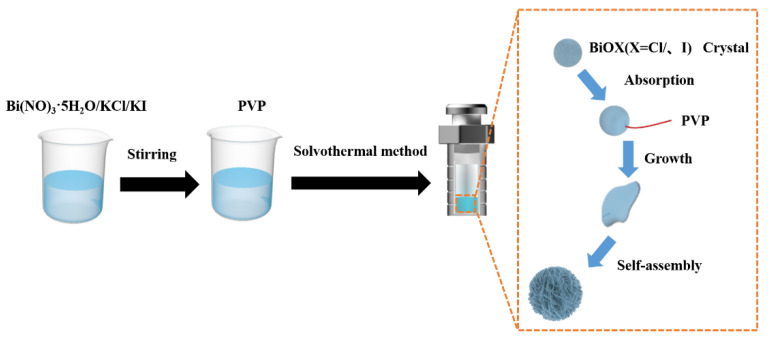
Procedures for preparation of 80%BiOCl/20%BiOI and its formation mechanism.

A facile solvothermal process was modified to prepare the 3D hierarchical porous ellipsoidal-shaped BiOX [26]. Firstly, 4 mmol of Bi(NO_3_)_3_.5H_2_O, 3.2 mmol of KCl, and 0.8 mmol of KI were dissolved in 21 mL of EG/DI water and the mixture was stirred until a clear solution was obtained. Then, PVP was added to the solution and stirred for another 10 min. The mixed solution was transferred into an autoclave (100 mL) and then heated in an oven at 150 °C for 12 h. After the oven was cooled to room temperature naturally, the synthesized powders were collected by centrifugation, and alternatively rinsed with deionized (DI) water and EG solution 5–10 times. Finally, these powders were dried at 80 °C for 12 h. The procedures were summarized in Scheme 1.

### 2.3. Characterization

Scanning electron microscopy (SEM, HITACHI, Tokyo, Japan) and transmission electron microscopy (TEM, Talos F200S, FEI Co., Oregon, Japan) were used to evaluate the microstructure morphology of the sample. The crystal structure of the sample was studied by X-ray diffraction spectroscopy (XRD, DMAX-UltimaIV, Rigaku Co., Tokyo, Japan). Surface chemical composition of samples studied by X-ray photoelectron spectroscopy (XPS, ESCALAB Xi+, Thermo Fisher, Waltham, MA, USA). The infrared absorption curve of the sample was characterized by Fourier transform infrared spectroscopy (FTIR, 6700, Nicolet, Madison, WI, USA). The optical absorption of the sample was measured by UV-vis diffused reflection spectroscopy (DRS, UV 3600 Plus UV-Vis, Shimadzu, Tokyo, Japan), and the photoluminescence behavior was studied by photoluminescence spectroscopy (PL, FLS980, Edinburgh Instruments, Livingston, UK). Photoelectric properties of samples studied by an electrochemical workstation (EIS, CS310H, Huakeputian Co., Beijing, China).

To characterize the photocatalytic performance of the sample, 50 mL of the aqueous solution was used for each organic dye, i.e., RhB, MO, MB, and MV. Except for MB aqueous solution which was prepared at a concentration of 20 mg/mL, the concentration of the other solutions was 50 mg/mL. For each photocatalytic performance test, 20 mg 80%BiOCl/20%BiOI powders were added and the solution was stirred for 1 h under the dark condition to homogeneously disperse the powders in the solution. The light source (Xenon light, 300 W) was then turned on to initiate the photocatalytic decomposition process. A small volume of the solution (5 mL) was taken out every 30 min, and the concentration of organic dyes was determined by the light absorption method. To reveal the main active species for the photocatalytic decomposition reactions, 20 mg of different kinds of radical scavenger or killer (EDTA as h^+^ radical scavenger, IPA as •OH^−^ radical scavenger, BQ as •O_2_^−^ radical scavenger, bought from Aladdin Co. (Shanghai, China)) were added into the original solutions. Then the above photocatalytic degradation tests were repeated for each kind of radical scavenger. The main active species was determined from the most effective radical scavenger.

## 3. Results and Discussions

### 3.1. SEM and TEM Analysis

**Figure 1:**
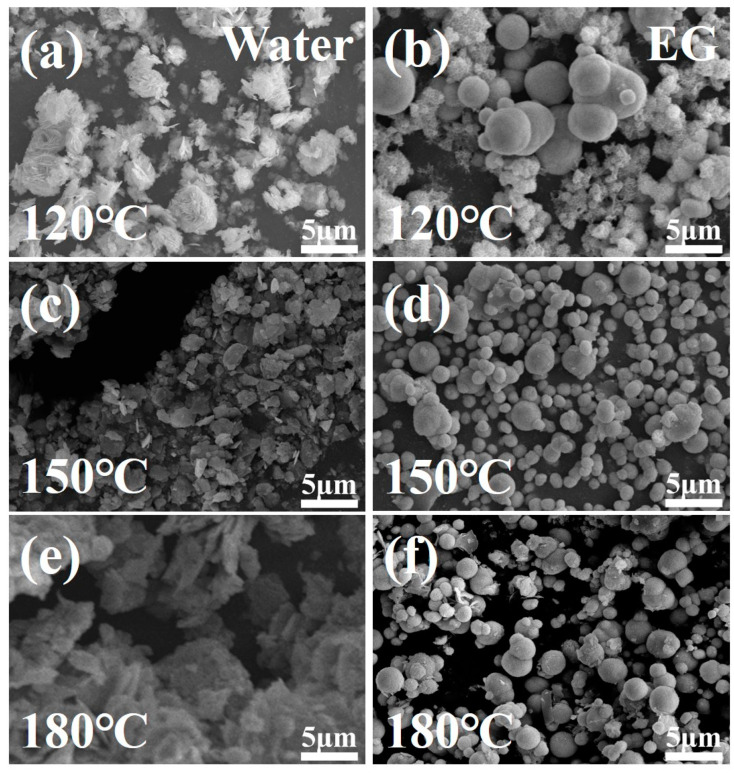
SEM images of 80%BiOCl/20%BiOI particles prepared at (**a**,**b**) 120 °C, (**c**,**d**) 150 °C and (**e**,**f**) 180 °C in water (**a**,**c**,**e**) and EG (**b**,**d**,**f**).

The effect of solvent and temperature on the morphology of 80%BiOCl/20%BiOI synthesized by the solvothermal method was first checked by preparing 80%BiOCl/20%BiOI with different solvents (DI water vs. EG) at different temperatures (120 °C, 150 °C, and 180 °C), and the results are shown in Figure 1. As shown in Figure 1a,c,f, when DI water was used as a solvent, the collected material exhibits an irregular shape composed of the stacking of micron-sized flakes with a thickness of ~100 nm. The size of the agglomerates was increased with elevated reaction temperature, as shown in Figure 1e. On the other hand, as can be seen in Figure 1b,d,e, spherical particles were obtained at all reaction temperatures when EG was used as a solvent. The SEM measurements show the average particle size was decreased from 3 μm at 120 °C to 1.5 μm at 150 °C, and then kept the same for the reaction temperature of 180 °C. Moreover, as shown in Figure 1b,e, the samples prepared at a temperature of 120 °C and 180 °C showed stacked irregular particles attached to the spherical particles. But the sample prepared at 150 °C was well-dispersed and showed much fewer irregular particles, as shown in Figure 1d. Therefore, the temperature for the following solvothermal process is set to 150 °C.

**Figure 2:**
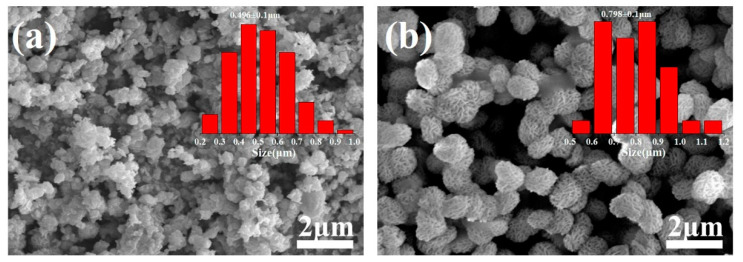
SEM images of 80%BiOCl/20%BiOI particles prepared by adding PVP into (**a**) water, and (**b**) EG at 150 °C.

**Figure 3:**
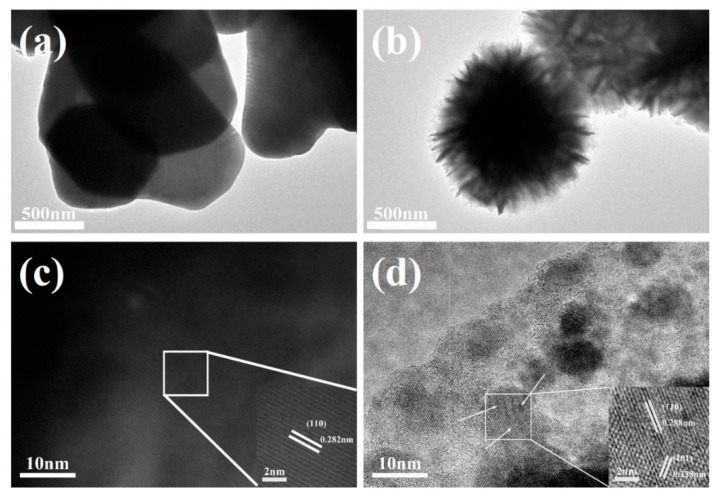
(**a**,**b**) TEM and (**c**,**d**) HRTEM images of 80%BiOCl/20%BiOI particles prepared by adding PVP into (**a**,**c**) water and (**b**,**d**) EG at 150 °C.

Figure 2 shows the SEM images of the samples prepared in the different solvents at 150 °C with the addition of PVP. As shown in Figure 2a, the sample synthesized by using an aqueous solution still shows irregular particles after the addition of PVP. But the average particle size was much smaller compared with that of the sample prepared under the same condition without the addition of PVP, and the dispersity of the particles was also greatly improved. The case is different when the solvothermal process proceeds in EG, the obtained particles are much more porous and exhibit an ellipsoidal shape after the addition of PVP (Figure 2b), which is similar to the previously reported flower-like BiOX particles [15,21,22]. The size distribution of the ellipsoidal particles is illustrated in the inset of Figure 3b, and the average particle size was determined to be 0.79 μm. Based on the above results, we proposed a process for the formation of the 80%BiOCl/20%BiOI porous ellipsoidal-shaped spheres. As shown in Scheme 1, Bi^3+^ first reacts quickly with Cl^−^ or I^−^ to form BiOX nanoplatelets, and then these nanoplatelets are self-assembled into microspheres under the influence of EG [27]. However, the size distribution of the formed BiOX microspheres is scattered, and some highly distributed free nanoplatelets are found on the surface of the BiOX microspheres. With the addition of PVP, the porous ellipsoidal BiOX particles were formed in uniform. It has been reported that PVP is a very important structure guiding agent [28]. Therefore, it is concluded that the porous and ellipsoidal microstructures were related to the adoption of PVP surfactant and EG solvent.

TEM is used to investigate the microstructure of 80%BiOCl/20%BiOI produced in EG and water, and the results are presented in Figure 3a,c and Figure 3b,d, which correspond to SEM images in Figure 2a and Figure 2b, respectively. Irregular particles (Figure 2a) are formed by the accumulation of sheets in water, as illustrated in the TEM picture in Figure 3a. The nanosheet is revealed in high-resolution TEM (HRTEM) images, as illustrated in Figure 3c. Regular lattice fringes in a large area were clearly evident, indicating the high crystallinity of the sample. The sample’s interplanar lattice spacing was measured to be 0.282 nm, which corresponds to the lattice spacing of BiOCl’s (110) plane [29]. From the TEM image shown in Figure 3b, it can be seen that the ellipsoidal-shaped particles are formed by closely stacked nanoplatelets. HRTEM images of the nanoplatelet are shown in Figure 3d. There are numerous clear lattice fringes visible, indicating that the nanoplatelets were well crystallized. The interplanar lattice spacing of the sample was measured to be 0.338 nm and 0.288 nm, which correspond to the lattice spacing of the (101) plane of BiOCl [29] and BiOI [23], respectively. This proves that our 80%BiOCl/20%BiOI sample is actually a mixed phase of BiOCl and BiOI. Another point worthy of note is that there exist many defects throughout the nanoplatelet as indicated by the arrows drawn in Figure 3d. However, proper defects are reported to have the ability to enhance the photocatalytic performance of BiOX material [30].

### 3.2. XRD Analysis

**Figure 4:**
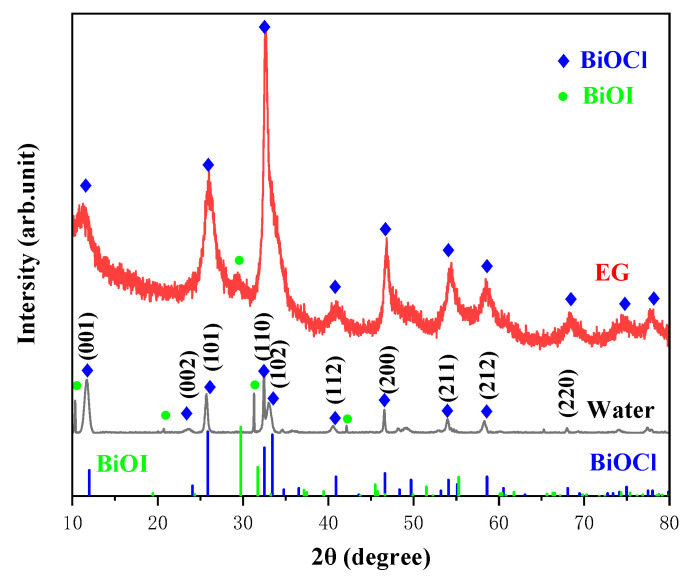
XRD patterns of 80%BiOCl/20%BiOI particles prepared by adding PVP into water and EG solvents.

XRD patterns of the as-prepared samples are shown in Figure 4. For the samples prepared in water with the addition of PVP, the collected XRD pattern is a sum of the diffraction peaks from BiOCl and BiOI, and no other diffraction peaks can be detected except for these two sets of XRD patterns. This further confirms that the as-prepared sample is a composite of BiOCl and BiOI in nature. For the sample prepared in EG with the addition of PVP, a similar diffraction pattern but with much broader peaks was observed, indicating that the crystallinity of the sample prepared in EG is poor with a small grain size. In addition, the average crystallite size of samples was calculated by Scherer’s equation [31,32]. The average crystallite sizes of the samples prepared in water and EG are 24.4 nm and 5.6 nm, respectively, which are consistent with the above analysis and HRTEM observations. 

### 3.3. XPS Analysis

**Figure 5:**
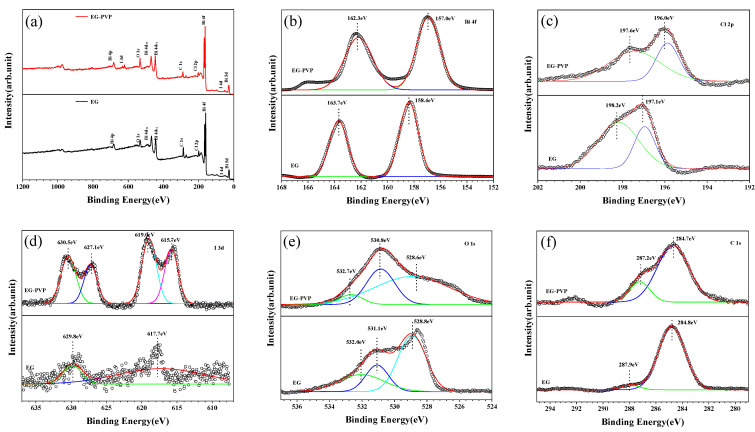
XPS survey spectra of 80%BiOCl/20%BiOI particles adding PVP into EG and without PVP in EG solvents (**a**), and high-resolution XPS spectra of Bi 4f (**b**); Cl 2p (**c**); I 3d (**d**); O 1s (**e**); C 1s (**f**).

The surface chemical compositions of EG and EG-PVP samples were measured by X-ray photoelectron spectroscopy. Figure 5b–d gives the corresponding Bi 4f, Cl 2p, I 3d, O 1s, and C 1s atomic orbitals and high-resolution spectra. As shown in Figure 5a, the characteristic peaks of Bi, Cl, I, O, and C were observed in the XPS measurement spectrum, indicating that the prepared samples contained these elements. Figure 5b shows that the Bi 4f spectrum can be fitted to the two characteristic peaks of Bi 4f_5/2_ and Bi 4f_7/2_, which are located at 163.7 eV and 158.4 eV, respectively. In contrast, the binding energy of the EG sample at the two positions of Bi 4f decreases, which may be due to the change in the chemical environment around the Bi atom after the addition of surfactant PVP [33]. Figure 5c shows the spectra of Cl 2p at 198.2 eV and 197.1 eV, which correspond to the characteristic peak of Cl^−^ in the material [34]. Similarly, the binding energy positions changed after the addition of surfactant PVP, indicating that the chemical environment around the Cl atom changed. It is worth noting that in Figure 5a, the characteristic peak of the I atom was detected in the EG-PVP sample, while the characteristic peak of the I atom was not detected in the EG sample. However, in the high-resolution spectra in Figure 5d, it is found that the 629.8 eV and 617.7 eV corresponding to the I 3d orbital of EG are the characteristic peaks of I 3d_3/2_ and I 3d_5/2_. In contrast, the EG-PVP sample has a slight shift from near the characteristic peak of I^−^, which may be due to the Coulomb coupling force between [Bmim]^+^ adsorbed on the surface of the sample and I^−^, resulting in the transfer of electrons from Bi to I [35]. The EG-PVP sample has two additional peaks (627.1 eV and 615.7 eV), which may be due to the existence of I in the sample in other forms besides the valence state of −1 [36]. Figure 5e shows the peak position near 528.6 eV, which is a typical characteristic peak of [Bi_2_O_2_]^2+^. For the peaks near 531 eV and 532 eV, it is due to the hydroxyl group in the sample preparation process [15]. Figure 5f shows that C has two peaks near 287 eV and 284 eV, which belong to the C-C bond and the C=O bond, respectively. This may be due to the adsorption of C-containing pollutants. In general, the surface chemical composition of the sample was analyzed by XPS, and the results showed that the surface contained Bi, O, Cl, and I elements.

### 3.4. FTIR Analysis

**Figure 6:**
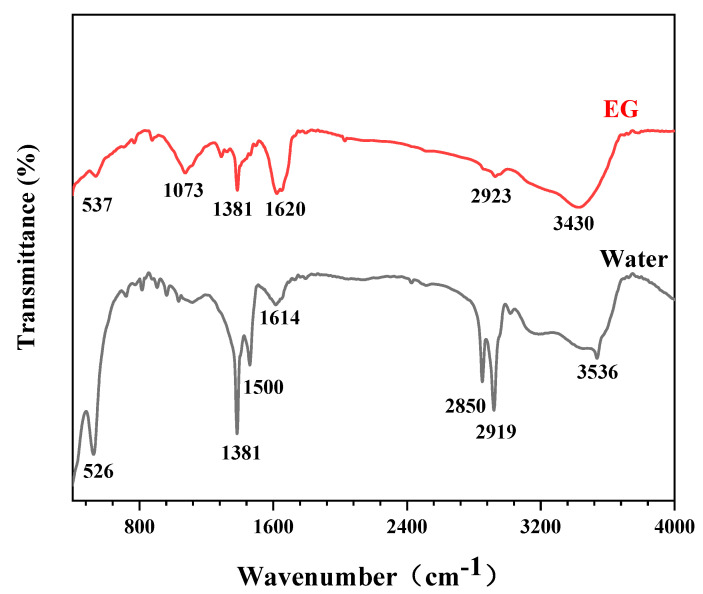
FTIR spectra of 80%BiOCl/20%BiOI particles prepared by adding PVP into water and EG solvents.

FTIR characterization was performed to reveal more information on the structural difference between the samples prepared in DI water and EG solutions with the addition of PVP, and the obtained results are summarized in Figure 6. Based on the previously published results, one can identify that the absorption band around 537 cm^−1^/526 cm^−1^ should be attributed to the Bi-O bond [37], and the broad band at 3430 cm^−1^/3536 cm^−1^ is related to the stretching vibration mode of the hydroxyl group –OH [24], and the 1620 cm^−1^/1614 cm^−1^ absorption peak stands for the bending vibration mode of –OH [36], which hints that both of the samples prepared in DI water and EG have surface formed –OH hydroxyl groups. The other bands are shown at around 2919 cm^−1^/2923 cm^−1^/2850 cm^−1^, and 1500 cm^−1^/1381 cm^−1^, which can be assigned to the surface attached to organic impurities [38,39,40,41]. Generally, the absorption bands for the sample prepared in EG are much broader than that of the sample prepared in DI water, which further confirms the poor crystallinity of the sample prepared in EG.

### 3.5. UV-Vis and PL Analysis

**Figure 7:**
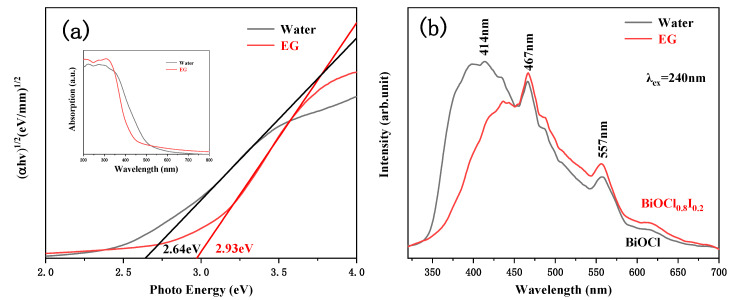
(**a**) UV-Vis absorption spectra (inset) and the corresponding (αhν)^1/2^ vs. photo energy (hν) curves, and (**b**) PL spectra of 80%BiOCl/20%BiOI particles prepared in water and EG solvents.

The optical bandgap of the sample was obtained through the UV-Vis absorption measurements. The results are shown in Figure 7a. As shown in the inset of Figure 7a, the absorption edges of the samples prepared in EG and DI water are positioned at about 430 nm and 500 nm, respectively. The optical bandgap can be extracted from the intercept of the Tauc plot of (αhν)^1/2^ vs. hν (α: absorption coefficient) on the abscissa axis [42,43,44,45], which gives values of 2.93 eV and 2.64 eV, respectively. The reduced optical bandgap value compared with that of pure BiOCl (3.2~3.3 eV) [17,45] may be originated from the incorporation of BiOI second phase in the sample, which possesses a much smaller bandgap (1.7~1.8 eV) [17,35]. It is well known that the specific band gap of BiOX largely depends on their microstructures, such as size, morphology, crystallinity, etc. [46] PL spectra can usually reveal information about the dynamics of photo-induced charge carriers [26,28,35,45]. The PL spectra excited at 240 nm are shown in Figure 7b. For the sample prepared in DI water, three distinct emission peaks can be clearly observed, i.e., a broad strong peak at 414 nm and two weak but sharp peaks at 467 nm and 557 nm. The sample prepared in EG shows similar emission peaks, but the dominant emission peak is changed to the one positioned at 467 nm. Furthermore, as shown in Figure 7b, the sample prepared in EG has reduced PL emission below 450 nm and comparable PL emission above 450 nm compared to the sample prepared in DI water. It has been commonly recognized that lower PL intensity indicates a lower recombination rate of photo-excited electron-hole pairs, which is beneficial for photocatalytic reactions [26]. Therefore, the 80%BiOCl/20%BiOI sample prepared in EG is expected to exhibit better photocatalytic performance.

### 3.6. Photoelectric Performance Analysis

**Figure 8:**
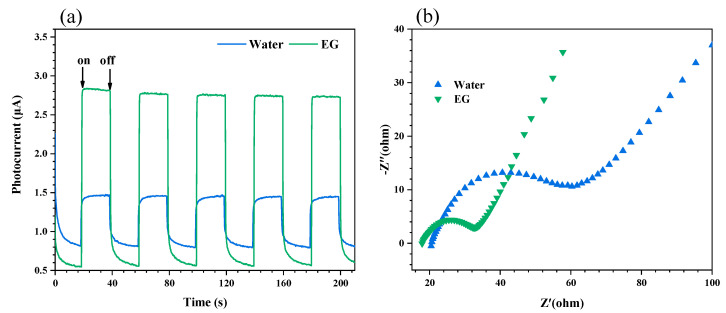
(**a**) Transient photocurrent response and (**b**) EIS spectra of 80%BiOCl/20%BiOI particles prepared by adding PVP into water and EG solvents.

The transient photocurrent response of an ITO electrode modified by the 80%BiOCl/20%BiOI sample is shown in Figure 8a when it is exposed to visible light. It can be seen that the photocurrent response of the sample prepared in EG is much larger than that of the sample prepared in water. This means that EG samples can generate more optical carriers under visible light irradiation. In the photocurrent response, it is worth noting how much current increases in the dark and light states, indicating the size of its photoelectric conversion capacity. Compared with dark current density, the photocurrent density of the sample prepared in EG and the sample prepared in water increased by 2 times and 5 times respectively, which indicated that the EG sample had better photoelectric conversion performance. The results of electrochemical impedance spectroscopy (EIS) are shown in Figure 8b. In general, the smaller the semicircle radius of the EIS Nyquist plot is, the smaller the resistance of the sample is. The radius of the sample prepared in EG is smaller than that prepared in DI water. This shows that EG samples have lower resistance and higher conductivity.

### 3.7. Photocatalytic Activity

**Figure 9:**
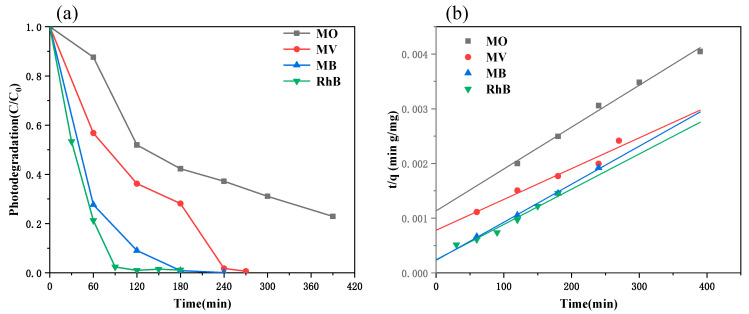
Photocatalytic degradation of RhB, MB, MV, and MO by the 80%BiOCl/20%BiOI microspheres (**a**), and the corresponding theoretical fittings based on the pseudo-first-order kinetics model (**b**).

The photocatalytic performance of the 80%BiOCl/20%BiOI sample prepared in EG was characterized by the degradation of various organic dyes (RhB, MO, MB, MV) under simulated sunlight, and the results are shown in Figure 9a. All of the tested organics can be gradually decomposed by 80%BiOCl/20%BiOI under illumination, and the photocatalytic degradation rate is highest for RhB and lowest for MO with the order of RhB > MB > MV > MO. As shown in Figure 9, 98% of RhB was degraded in 90 min and 90.4% of MB was degraded in 120 min. As the ratio of dyes to catalyst decreases, the degradation process becomes slower. From the kinetic data, this was a typical adsorption process. The degradation process was fitted to a second-order kinetic model: $\frac{t}{q}=\frac{1}{k_{2}q_{e}{}^{2}}+\frac{t}{q_{e}}$. where *k*_2_ (g/mg per minute) is the rate constant of second-order adsorption and *q* and *q*_e_ (mg/g) are the amounts of species adsorbed on the adsorbent at time *t* and at equilibrium, respectively [47,48,49]. The linear relationship, as reported in Figure 9b, indicates that the second-order kinetics is applicable, and the half-life of the process can be calculated as: $t_{0.5}=\frac{1}{k_{2}q_{e}}$. The *k*_2_, *q*_e_, and *t*_0.5_ parameters for the degradation process are presented in Table 1. In addition, the photocatalytic efficiency of the 80%BiOCl/20%BiOI sample was compared to the previously reported articles under visible light irradiation, and shown in Table 2, both degradation time and efficiency exhibit good performance.

### 3.8. Photodegradation Mechanism

**Figure 10:**
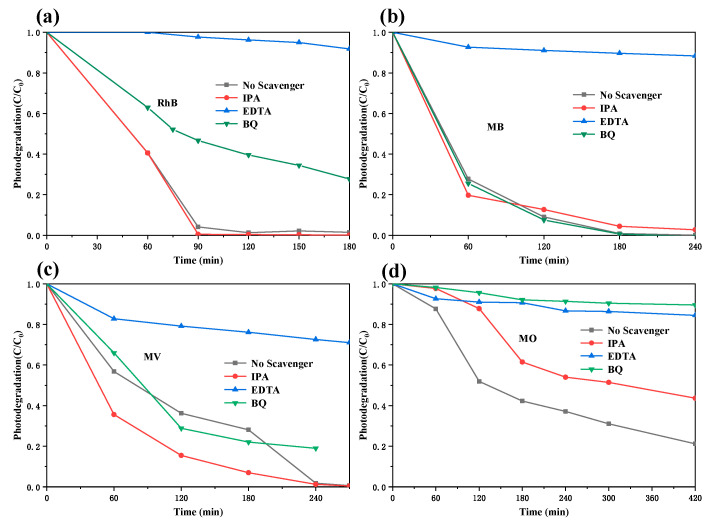
Photocatalytic degradation of (**a**) RhB, (**b**) MB, (**c**) MV, and (**d**) MO by the 80%BiOCl/20%BiOI microspheres in the presence of different radical scavengers.

To reveal the active species for the photocatalytic degradation of each tested organics, various radical scavengers were added to the test solution and their effects on the photocatalytic performance are shown in Figure 10. 

For RhB, the addition of hole scavenger (EDTA) almost completely impedes the degradation process while the hydroxyl radical scavenger (IPA) has no obvious effects on the photocatalytic reaction, and the superoxide scavenger (BQ) only hindered the degradation reaction to some extent. After 180 min of photocatalytic reaction, 8.2%, 72.3%, and 99.9% of RhB were degraded with the addition of EDTA, BQ, and IPA, respectively. Alternatively, 98.5% of RhB was decomposed after 180 min without the addition of any radial scavengers, as shown in Figure 10a. These results indicate that the major active species for the photocatalytic degradation of RhB are photogenerated holes (h^+^), and the minor active species are superoxide radicals (•O_2_^−^) formed under illumination. The hydroxyl radical (•OH^−^) is insignificantly involved in the degradation process of RhB. 

Similarly, the addition of EDTA is also very effective in preventing the degradation of MB, and the other two types of radical scavengers have no detectable effects on the photocatalytic degradation of MB. After 240 min of photocatalytic reaction, 11.6%, 100%, and 97.% of MB were degraded with the addition of EDTA, BQ, and IPA, respectively. Alternatively, 99.9% of MB was decomposed after 240 min without the addition of any radial scavengers, as shown in Figure 10b. It proves that photogenerated holes (h^+^) are the main active species for the photocatalytic degradation of MB. 

For the degradation of MV, the addition of EDTA also suppressed the photocatalytic reaction significantly, but not as much as in the cases of RhB and MB. The addition of BQ also has an insignificant effect on the degradation process of MV. However, IPA seems to speed up the photocatalytic degradation of MV, which cannot be simply interpreted by the inhibited recombination of the photoinduced electrons and holes [16] and needs further investigation. After 240 min of photocatalytic reaction, 28.9%, 81.0%, and 99.5% of MV were degraded with the addition of EDTA, BQ, and IPA, respectively. Alternatively, 99.4% of MV was decomposed after 240 min without the addition of any radial scavengers, as shown in Figure 10c.

For the degradation of MO, both EDTA and BQ can greatly hinder the degradation process while the IPA can also suppress the photocatalytic reaction. After 420 min of photocatalytic reaction, 15.5%, 10.4%, and 56.3% of MO were degraded with the addition of EDTA, BQ, and IPA, respectively. Alternatively, 78.8% of MO was decomposed after 420 min without the addition of any radial scavengers, as shown in Figure 10d. Such results indicate that photogenerated holes (h^+^) and superoxide radicals (•O_2_^−^) play a dominant role in the degradation of MO, and hydroxyl radical (•OH^−^) is the minor active species for the photocatalytic reaction. Based on the above results, one can find that photogenerated holes (h^+^) are always playing the most important role in the photocatalytic reactions of various organic dyes when 80%BiOCl/20%BiOI is used as the catalyst.

Li et al. proposed that in the BiOCl/BiOI sample, BiOI would produce photoelectrons and then generate holes on its surface after being irradiated by visible light, while BiOCl would accept electrons and react with oxygen at the interface to produce superoxide radicals (•O_2_^−^) [59,60,61]. Due to the low BiOI content in this study, there are fewer electrons. For the degradation process of RhB, fewer electrons are difficult to produce enough superoxide radicals (•O_2_^−^), which shows that the effect of photogenerated holes (h^+^) is greater than that of the superoxide radicals (•O_2_^−^). For MO, there are fewer superoxide radicals (•O_2_^−^) required in the degradation process, so the effect of superoxide radicals (•O_2_^−^) is similar to that of photogenerated holes (h^+^). For MB and MV, their degradation processes are almost only related to photogenerated holes (h^+^).

## 4. Conclusions

Porous ellipsoidal-shaped 80%BiOCl/20%BiOI microspheres with a particle size of 0.79 μm and crystallite size of 5.6 nm were prepared through a facile solvothermal method by using EG as a solvent, in which process the addition of PVP is demonstrated to be crucial to serve as a templating agent. The effect of types of solvents and process temperature on the microstructure of the final product was also investigated. XRD characterization results show that the obtained 80%BiOCl/20%BiOI is a composite of BiOCl and BiOI in nature. The photocatalytic performance of the 80%BiOCl/20%BiOI microspheres was tested through the degradation of various organic dyes, i.e., RhB, MO, MV, and MB, under illuminated conditions. It was found that 98% of RhB can be most efficiently degraded in 90 min by 80%BiOCl/20%BiOI and the degradation rate for the tested organic dyes follows the order of RhB > MB > MV > MO. With the help of radical scavengers, the main active species for the photocatalytic degradation of each organic dye were studied, and photogenerated holes (h^+^) were found to be the main active species in all cases. Our results show that the porous ellipsoidal-shaped 80%BiOCl/20%BiOI is easy to prepare and promising for photocatalytic degradation of organic pollutants. In future research, the degradation performance of the material under real environmental pollution such as different PH, temperature, and pollutant concentrations will be further improved.

## Figures and Tables

**Table 1:** Kinetic date of the photodegradation process.

Dyes	K_2_ (g/mg min)	q_e_ (mg/g)	T_0.5_ (min)
MO	1.51 × 10^13^ ± 5.38 × 10^11^	1.31 × 10^5^ ± 6.44 × 10^4^	148.1 ± 19.87
MV	4.05 × 10^13^ ± 4.27 × 10^12^	1.78 × 10^5^ ± 1.99 × 10^5^	138.6 ± 35.9
MB	8.96 × 10^13^ ± 8.99 × 10^12^	1.44 × 10^5^ ± 4.49 × 10^4^	33.2 ± 6.01
RhB	9.99 × 10^13^ ± 1.35 × 10^13^	1.55 × 10^5^ ± 1.33 × 10^5^	37.5 ± 12.9

**Table 2:** Comparison of degradation performance for various photocatalysts under visible light irradiation.

S. No.	Sample	Dyes	Time (min)	Efficiency (%)	Reference
1	Bi_2_O_3_/g-CN	RhB	180	~83	[50]
2	10 wt% Ag/CeVO_4_/g-C_3_N_4_	MB	120	~94	[51]
3	ZnAl_1.98_Ce_0.02_O_4_	MB	240	72.5	[52]
4	TiO_2_–graphene	MB	100	98.8	[53]
5	Fe_2_O_3_/Cu_2_O nanomaterial	RhB	120	79.15	[54]
6	Zn doped WO_3_	MB	120	78	[55]
7	6 wt% CdS/BiOCl	RhB	**	78	[56]
8	MoS_2_/g-C_3_N_4_	RhB	420	~90	[57]
9	SnO_2_ nanoparticles	MB	240	~96	[58]
10	80%BiOCl/20%BiOI	RhB	90	98	This
		MB	120	90.4	work

**: no data.

## Data Availability

All data generated or analyzed during this study can be available from the corresponding author on request.
